# Feasibility, Engagement, and Usability of a Remote, Smartphone-Based Contingency Management Program as a Treatment Add-On for Patients Who Use Methamphetamine: Single-Arm Pilot Study

**DOI:** 10.2196/47516

**Published:** 2023-07-06

**Authors:** Kevin A Hallgren, Mark H Duncan, Matthew D Iles-Shih, Eliza B Cohn, Connor J McCabe, Yanni M Chang, Andrew J Saxon

**Affiliations:** 1 Department of Psychiatry and Behavioral Sciences University of Washington Seattle, WA United States; 2 Center of Excellence in Substance Addiction Treatment and Education Veterans Health Administration Puget Sound Health Care System Seattle, WA United States

**Keywords:** contingency management, methamphetamine use disorder, mobile health, mHealth, mobile phone

## Abstract

**Background:**

In the United States, methamphetamine-related overdoses have tripled from 2015 to 2020 and continue to rise. However, efficacious treatments such as contingency management (CM) are often unavailable in health systems.

**Objective:**

We conducted a single-arm pilot study to evaluate the feasibility, engagement, and usability of a fully remotely delivered mobile health CM program offered to adult outpatients who used methamphetamine and were receiving health care within a large university health system.

**Methods:**

Participants were referred by primary care or behavioral health clinicians between September 2021 and July 2022. Eligibility criteria screening was conducted by telephone and included self-reported methamphetamine use on ≥5 out of the past 30 days and a goal of reducing or abstaining from methamphetamine use. Eligible participants who agreed to take part then completed an initial *welcome phase* that included 2 videoconference calls to register for and learn about the CM program and 2 “practice” saliva-based substance tests prompted by a smartphone app. Participants who completed these welcome phase activities could then receive the remotely delivered CM intervention for 12 consecutive weeks. The intervention included approximately 24 randomly scheduled smartphone alerts requesting a video recording of themselves taking a saliva-based substance test to verify recent methamphetamine abstinence, 12 weekly calls with a CM guide, 35 self-paced cognitive behavioral therapy modules, and multiple surveys. Financial incentives were disbursed via reloadable debit cards. An intervention usability questionnaire was completed at the midpoint.

**Results:**

Overall, 37 patients completed telephone screenings, with 28 (76%) meeting the eligibility criteria and consenting to participate. Most participants who completed a baseline questionnaire (21/24, 88%) self-reported symptoms consistent with severe methamphetamine use disorder, and most had other co-occurring non-methamphetamine substance use disorders (22/28, 79%) and co-occurring mental health disorders (25/28, 89%) according to existing electronic health records. Overall, 54% (15/28) of participants successfully completed the welcome phase and were able to receive the CM intervention. Among these participants, engagement with substance testing, calls with CM guides, and cognitive behavioral therapy modules varied. Rates of verified methamphetamine abstinence in substance testing were generally low but varied considerably across participants. Participants reported positive opinions about the intervention’s ease of use and satisfaction with the intervention.

**Conclusions:**

Fully remote CM can be feasibly delivered within health care settings lacking existing CM programs. Although remote delivery may help reduce barriers to treatment access, many patients who use methamphetamine may struggle to engage with initial onboarding. High rates of co-occurring psychiatric conditions in the patient population may also contribute to uptake and engagement challenges. Future efforts could leverage greater human-to-human connection, more streamlined onboarding procedures, larger incentives, longer durations, and the incentivization of non–abstinence-based recovery goals to increase uptake and engagement with fully remote mobile health–based CM.

## Introduction

### Background

The United States is facing a rapidly growing methamphetamine use disorder epidemic, with fatal overdoses involving methamphetamine more than quadrupling from 2015 to 2021 [1]. In Washington state, the rate of fatal overdoses involving methamphetamine tripled from 2015 to 2021 and is approaching the rate of fatal overdoses involving fentanyl and other opioids [2]. Owing to the country’s ongoing opioid and methamphetamine use disorder epidemics, drug overdose death rates remain at an all-time high, with >100,000 deaths from drug overdoses in 2021 [3].

Increasing access to evidence-based treatments for methamphetamine use is vital for mitigating the overdose crisis. Medications approved by the US Food and Drug Administration (FDA) are available and can be prescribed to treat opioid use disorder, but there are currently no medications approved by the FDA for methamphetamine use disorder. Contingency management (CM) is an efficacious and well-studied behavioral intervention for stimulant use disorders that offers financial rewards when patients can verify recent abstinence from methamphetamine on substance tests [4]. By rewarding abstinence, CM aims to leverage the brain’s reward system to help individuals stop using substances by increasing incentives for drug abstinence. However, access to CM is not yet widely available in most health care settings [5,6].

CM has been challenging to implement in some settings (eg, primary care), in part because of limited staffing and challenges with integration into clinical workflows [7,8]. In addition, some patients may have difficulty accessing in-person CM services; for example, some CM programs may require patients to visit clinics multiple times per week to complete substance tests and receive financial incentives. These barriers were further compounded by the COVID-19 pandemic, which hindered access to in-person services and required many clinical settings to prioritize other time-sensitive issues (eg, changes in policies and practices related to the pandemic) [9].

Mobile health (mHealth) technology shows promise in aiding the delivery of CM in health care settings. For example, digital apps and smartphone cameras could make it easier for patients to submit biological substance self-testing results (eg, patients may video record themselves completing saliva-based substance tests from any location without requiring in-person clinic visits or clinician time to administer the substance tests). Similarly, these technologies can make it easier to disburse financial incentives after recent methamphetamine abstinence has been verified via self-testing (eg, money can be loaded immediately onto reloadable debit cards by staff who can review videos from any location) [10-13]. Commercially available mHealth programs could also potentially aid the delivery of CM in health care settings where clinical expertise, staffing, and workflow limitations have traditionally hindered the implementation of CM, for example, by delegating the responsibilities for verifying methamphetamine abstinence and disbursing rewards to dedicated CM program staff outside the clinic.

### Objectives

There is a need for a better understanding of how mHealth-based CM programs can be made available to patients in existing outpatient clinical settings. To understand this, we conducted a single-arm pilot study that aimed to evaluate the feasibility of offering a commercially available mHealth CM program to patients who used methamphetamine and were receiving treatment within a health system without existing CM programming. For this pilot, the commercially available mHealth program was modified according to input from clinicians in the participating clinics (described in the following sections). The primary outcomes for the pilot study focused on evaluating the *uptake* of the program (ie, how often participants initiated the intervention), *engagement* with the program (ie, for participants with successful uptake, how often they engaged with the core components of the intervention), and *usability* (ie, for participants who received the intervention, whether they perceived the intervention as easy to use, satisfactory, and useful). This pilot study was not a randomized trial designed to assess the efficacy or effectiveness of the intervention. However, this study’s evaluation of uptake, engagement, and usability can provide insights into ways to improve the delivery of mHealth-based CM interventions. Therefore, the aim of this formative research was to provide insights into the feasibility, engagement, and usability of mHealth-based CM offered to patients who use methamphetamine, which could support the designs of future, larger-scale effectiveness and implementation studies with similar populations.

## Methods

### Setting and Sample

This was a single-arm pilot study of patients who were offered a completely remotely delivered mHealth-based CM program aimed at helping participants reduce or abstain from methamphetamine use. Participants were receiving outpatient care from 1 of 7 primary care clinics or from 1 specialty substance use disorder clinic. All participating clinics were part of a large academic health system in Washington state, United States. The health system primarily served patients in urban and suburban areas. Several clinics were located within a safety net medical center that serves many patients who are unhoused or have limited resources. The inception of the study was driven largely by feedback from clinicians within the health system who expressed a strong desire to offer CM to patients who use methamphetamine given the lack of FDA-approved medications for methamphetamine use disorder and the lack of existing CM programs within their clinical setting. Before launching the study, the research team met with clinicians in several participating clinics to solicit their input on how the study goals and procedures could fit with their understanding of their clinics’ workflows and needs related to methamphetamine use treatment.

The study recruitment period was open from September 2021 to July 2022. The staff at participating clinics were provided with information about the study and recruitment flyers that they could distribute to their patients whom they believed may benefit from the intervention. Patients could call the study phone number printed on the flyer to complete a telephone eligibility screening. Alternatively, providers could refer their patients to the study through a secure message in the electronic health record (EHR) system if patients provided verbal consent to be contacted by the research team. In these cases, the research team contacted the patient after receiving a secure EHR message from the clinician to assess eligibility.

Eligibility was assessed during a telephone screening with a university-based research coordinator. Eligibility criteria included (1) receiving care from a participating clinic, (2) self-reported methamphetamine use for ≥5 out of the past 30 days, (3) self-reported goal to reduce or abstain from methamphetamine use, (4) age of ≥18 years, and (5) ability to read and communicate in English per self-report. Smartphone ownership was not an eligibility requirement as the study provided smartphones and data plans to participants who did not have them. Patients who were eligible and interested in participating in the study provided written informed consent via e-signature and were emailed a link to complete the baseline questionnaires described in the following sections. Following informed consent, participants were scheduled with an intake appointment to initiate the “welcome phase” of the DynamiCare program (described in the following sections).

The study aimed to recruit up to 30 participants—a sample size that was deemed adequate for obtaining preliminary information about feasibility, engagement, and usability based on a previous pilot study conducted in one of the participating clinics that had a similar sample size [14] and based on consensus among the research team.

### Ethics Approval

All study procedures were approved by the University of Washington Institutional Review Board (STUDY00013066). All research and intervention procedures were completed remotely via telephone, videoconference, SMS text message, and the smartphone app.

### Intervention

#### Overview

Participants in this pilot study received access to a brief version of the DynamiCare Health program (Boston, Massachusetts) that was customized for this study. The program is commercially available and has been evaluated as a tool for increasing nicotine cessation in people who smoke or vape nicotine [15-17], alcohol abstinence in adults with alcohol use disorder [10], and drug abstinence and treatment adherence in patients with opioid use disorders [11-13]. No studies have been published regarding the feasibility of these particular program components when used as a completely remote intervention for methamphetamine use. The elements of the intervention were customized for this study, including the duration of the intervention, the requirements and values for CM rewards, and the specific cognitive behavioral therapy (CBT) modules that were offered. In addition, the procedures for referring and onboarding patients to the intervention were modified based on input from clinicians during initial stakeholder meetings, primarily to minimize disruptions to clinical workflows. For example, with DynamiCare’s more usual onboarding process, referring clinicians typically provide patients with a brochure and reloadable debit card during the referral stage so that rewards are received more immediately. However, in this study, debit cards were sent by mail during the welcome phase as it was believed by the investigators and partnering clinicians to be more feasible in the settings where the study took place.

#### Welcome Phase

Before receiving the intervention, participants were required to complete a “welcome phase,” during which they obtained study materials (eg, downloaded the smartphone app and received substance testing supplies and a reloadable debit card by mail) and received instructions on how to participate in the program. To complete the welcome phase, participants were asked to complete 2 videoconference calls that outlined the program structure, provided instructions on how to complete the intervention tasks (eg, how to activate and use the reloadable debit card and how to video record themselves taking the saliva-based substance tests through the smartphone app), and set up the tailored aspects of the program (eg, identify typical times of the day at which they were awake and could be prompted by the smartphone app to complete substance tests). To complete the welcome phase, participants were also asked to video record themselves completing 2 “practice” substance tests within 6 hours of being prompted to do so by the smartphone app. Video recordings were reviewed by DynamiCare staff to visually confirm the identity of the participant in the video, ensure that the test was taken properly, confirm that the test result was clearly visible, record the test result, and disburse any rewards that were earned. Participants received US $10 for each videoconference and US $5 for each practice test (regardless of the results of the test) during the welcome phase. Rewards were disbursed onto a reloadable debit card with features aimed at reducing the potential risk of substance use (eg, blocked for spending at bars and liquor stores).

#### Primary Intervention

Participants who completed the welcome phase activities could then receive the primary mHealth intervention for up to 12 weeks. The intervention included randomly timed prompts from the smartphone app to complete saliva-based substance tests approximately twice per week, with an US $8.42 incentive if the result indicated recent abstinence from methamphetamine and other amphetamines. For participants who were prescribed an amphetamine, the financial incentive was only contingent on the substance test results being negative for methamphetamine. For all participants, CM rewards were not contingent on abstinence from drugs other than methamphetamine and other amphetamines (eg, cocaine abstinence was not rewarded), a decision that was informed by input from clinicians in participating clinics. For participants with continuous streaks of abstinence from methamphetamine and other amphetamines, the value of the incentive increased, and the frequency of testing decreased with each consecutive substance test (see Multimedia Appendix 1 [18-23] for detailed testing and reward schedules). Participants were mailed saliva-based OralTox Oral Fluid Drug Tests (Premier Biotech) that included separate panels testing for methamphetamine (cutoff of 50 ng/mL) and other amphetamines (cutoff of 50 ng/mL), as well as other substances that were not factored into the CM rewards.

During the intervention, participants were also given access to 35 brief, self-paced CBT modules. Participants received a US $1 reward for each CBT module they completed. Participants were prompted to complete up to 9 surveys through the smartphone app, with a US $1 or US $2 reward for each survey that was completed.

Throughout the intervention, participants had access to a CM guide with lived experience of recovery from a substance use disorder. The CM guide provided encouragement and support for participants’ treatment goals of abstaining or reducing methamphetamine use and encouraged participants to use various components of the mHealth program to help them achieve their goals. All participants were encouraged to meet with the CM guide once per week. Initially, participants were not rewarded for meeting with the CM guide. However, to increase engagement, rewards were offered for meetings with CM guides for participants who completed the welcome phase in March 2022 or later, with these participants being eligible to receive US $20 for their first meeting with the CM guide and US $10 each week they met with the CM guide thereafter (ie, up to US $140 for meetings with CM guides). In total, participants who completed the welcome phase before March 2022 (n=5) were eligible to receive up to US $280 for completing all aspects of the intervention plus another US $45 for completing the research assessments (described in the following sections; up to US $325 total). Participants who completed the welcome phase in March 2022 or later (n=10) were eligible to receive up to US $420 for completing all aspects of the intervention plus another US $45 for completing the research assessments (up to US $465 total).

Referring clinicians were addiction, mental health, and medical service providers and were offered access to a web-based dashboard where they could review their patients’ progress in the intervention (eg, results of substance tests, CBT modules completed, and values of rewards disbursed). On the basis of input from clinicians during initial stakeholder meetings, clinicians were also sent monthly updates summarizing their patients’ progress in the intervention via secure messages.

### Measures

#### Overview

Participants were asked to complete research questionnaires through the University of Washington REDCap (Research Electronic Data Capture; Vanderbilt University) system [24] after enrollment in the study (baseline). For participants who completed the welcome phase and were therefore eligible to receive the intervention, additional surveys were requested at the 6-week (middle of intervention) and 12-week (after the intervention) time points. Participants received US $10 incentives for each completed research questionnaire plus US $15 for completed qualitative interviews that are not analyzed in this paper but will be reported in a future qualitative study.

#### Demographics, Socioeconomic Factors, and Substance Use at Baseline

Demographics (age, race, ethnicity, gender, and marital status) were assessed in the baseline questionnaire. If participants did not complete the baseline questionnaire, this information was obtained from the EHR.

Socioeconomic factors including education, current employment, housing insecurity, financial insecurity, access to transportation, and past-year criminal justice system involvement were assessed in the baseline questionnaire using questions from the Protocol for Responding to and Assessing Patients’ Assets, Risks, and Experiences [25].

Past–30-day methamphetamine use and methamphetamine treatment goals (abstinence or reduced use) were assessed via self-report during the initial telephone screening. The baseline questionnaire further asked participants to report the routes through which they consumed methamphetamine (eg, smoking, oral ingestion, and injection) and asked participants to report other drugs they had used in the previous 30 days [26]. The baseline questionnaire included a Substance Use Symptom Checklist [18] in which participants self-reported the presence or absence of each of the 11 criteria for methamphetamine use disorder according to the Diagnostic and Statistical Manual of Mental Disorders, Fifth Edition (DSM-5), over the past year. The number of criteria endorsed was then summed and categorized to reflect symptoms consistent with mild (2-3 criteria), moderate (4-5 criteria), or severe (6-11 criteria) methamphetamine use disorder in alignment with the DSM-5 [19]. Receipt of medications for opioid use disorder (eg, buprenorphine) and prescribed amphetamines (eg, for attention-deficit/hyperactivity disorder) was assessed during the screening call and confirmed via the EHR. Co-occurring substance use and mental health disorders diagnosed within the university health system during the year before study enrollment were extracted from the EHR using bulk electronic extraction [27].

#### Engagement

Patients could interact with the intervention in multiple ways, and therefore, engagement could not be fully captured using a single measure. We derived multiple engagement measures to characterize the different ways in which participants interacted with the intervention using data generated through the mHealth app and program records. We defined *uptake* as the percentage of participants who completed the welcome phase out of those who consented to participate in the study. We defined *early withdrawal from the program* as the percentage of participants who received the intervention and requested to terminate the intervention before the 12-week period was over. We also described the *number of substance tests completed* and the *number of substance tests indicating recent methamphetamine and amphetamine abstinence* (out of all tests prompted and, alternatively, out of all tests completed), the *number of CM guide calls completed*, the *number of CBT modules completed*, and *total incentives earned* for completing the intervention and research activities.

#### Usability

Usability was measured using a modified mHealth App Usability Questionnaire [28] that was administered during the sixth week of the intervention. The questionnaire included items that asked participants to rate their level of agreement or disagreement with statements reflecting the intervention’s ease of use (eg, “It was easy for me to use the program”), satisfaction with the intervention (eg, “I would use this program again”), and the usefulness of the intervention (eg, “The program helped me manage my substance use effectively”). Response options were on a 7-point Likert scale ranging from *strongly disagree* to *strongly agree*. In total, 2 additional questions asked participants to rate their thoughts on the amount of time required to participate in the program on a day-to-day basis and their satisfaction with the 3-month duration of the pilot intervention. Response options for these questions were on a 5-point Likert scale ranging from “much too long” to “much too short.”

#### Supplemental Clinical Outcomes

Additional questionnaires were completed at the baseline and 12-week assessments to gauge potential changes in clinical outcomes. However, these measures were considered supplemental to this study given that the single-arm pilot study was neither designed nor powered to test the impact of the intervention on clinical outcomes. The supplemental measures are described in more detail in Multimedia Appendix 1 and include measures of (1) methamphetamine use disorder severity, (2) depression symptoms, (3) methamphetamine abstinence self-efficacy, and (4) social support.

### Analytic Plan

Descriptive analyses were used to characterize participant flow, including the number of participants who completed telephone screenings, informed consent, and the research questionnaires at baseline and follow-ups. Descriptive analyses (number and percentage) were also used to characterize participant demographics, socioeconomic factors, substance use at baseline, and co-occurring substance use and mental health disorders, including for the full sample of participants who consented to take part in the study and for subgroups of participants who completed and did not complete the welcome phase. Differences between these subgroups were tested using Fisher exact tests.

The engagement metrics were characterized descriptively. The uptake measure was quantified as the number and percentage of participants who completed the welcome period and had access to the intervention (out of those who consented to participate in the study). The remaining engagement measures were characterized using means, SDs, medians, ranges, and IQRs. These descriptive statistics were computed to describe both the central tendency and distributions and variability in engagement with the intervention’s different components and were computed within the nested subsample of participants who completed the welcome phase and, therefore, had access to the primary intervention. Usability measures were characterized descriptively by quantifying the number of participants who reported agreement, disagreement, or neutrality with statements reflecting the intervention’s usability among those who completed the welcome phase (and, thus, had access to the primary intervention) and the usability questionnaire.

## Results

### Participant Flow and Uptake in the Intervention

**Figure 1:**
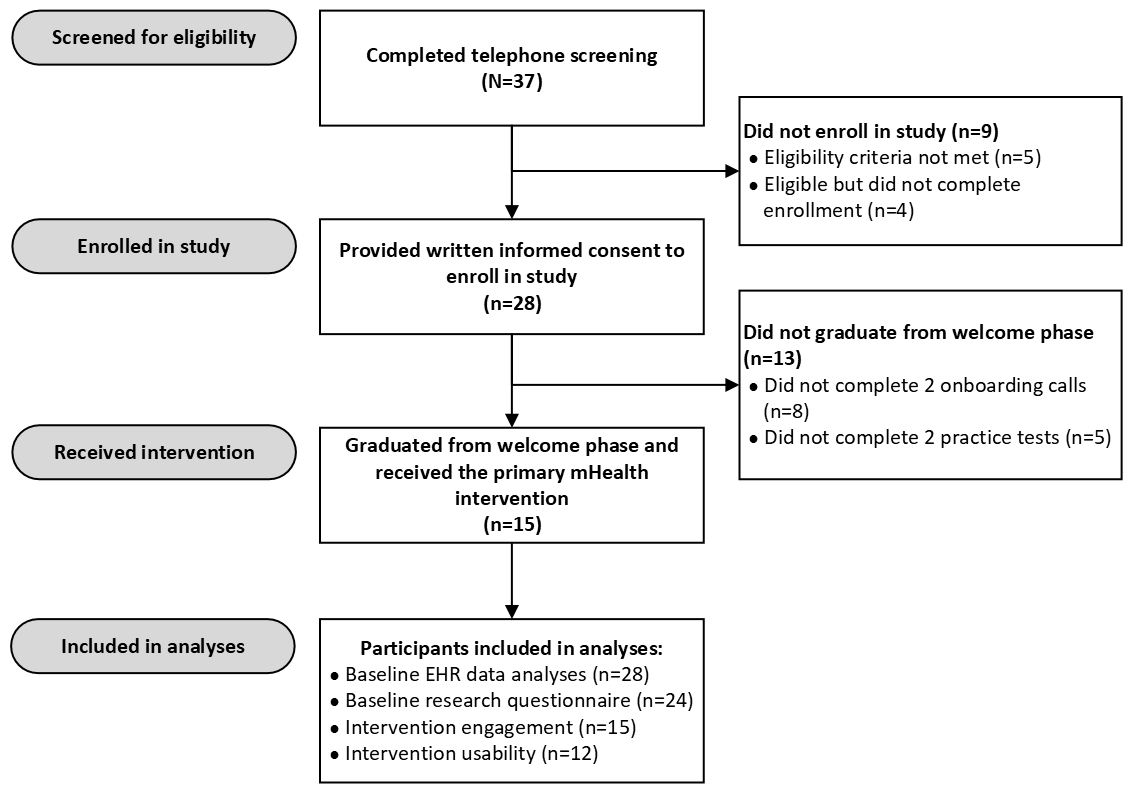
Participant flow through screening, enrollment, and receipt of the mobile health (mHealth) intervention. Baseline electronic health record (EHR) data analyses included all participants who consented to take part in the study. Baseline research questionnaire analyses included all participants who consented to take part in the study and completed the baseline research questionnaire. Intervention engagement analyses included all participants who received the intervention. Intervention usability analyses included participants who received the intervention and completed the midintervention usability questionnaire.

Participant flow is depicted in Figure 1. A total of 37 patients completed the telephone screening, of whom 5 (14%) were ineligible for the study and 4 (11%) were eligible but did not complete the informed consent process. The remaining 76% (28/37) of individuals were eligible for the study and provided written informed consent to participate via e-signature. Of these 28 individuals, 25 (89%) made contact with the mHealth app vendor, 15 (60%) of whom completed the welcome phase and, therefore, received access to the primary intervention (ie, 15/28, 54% uptake among all individuals who were eligible and provided informed consent). These participants took a mean of 34.07 (SD 13.86) days to fulfill all steps to complete the welcome phase (note that this mean and SD exclude an outlier, reflecting a participant who initially expressed a desire to stop taking part in the welcome phase and then resumed the program and completed the welcome phase 5 months later).

### Demographics, Socioeconomic Factors, and Substance Use

Of the 28 enrolled participants, 22 (79%) were recruited from primary care clinics and 6 (21%) were recruited from the specialty substance use disorder clinic. A total of 7% (2/28) of the enrolled participants did not own smartphones and were provided with phones and data plans by the study.

Table 1 provides baseline descriptive statistics for measures that were available for all enrolled participants as well as for subgroups who did and did not complete the welcome phase. As shown in Table 1, half (14/28, 50%) of the participants were aged 46 to 64 years. Most participants (23/28, 82%) were men. Participants were 4% (1/28) American Indian or Alaska Native; 11% (3/28) Black or African American; 14% (4/28) Hispanic, Latino, or Latina or of Spanish origin; 4% (1/28) Middle Eastern or North African; 57% (16/28) White; and 11% (3/28) multiracial. Most participants (22/28, 79%) were single, divorced, or widowed. Many (11/28, 39%) belonged to sexual orientation minority groups (7/28, 25% gay; 3/28, 11% bisexual; and 1/28, 4% lesbian). Most (20/28, 71%) reported using methamphetamine on ≥15 out of the past 30 days, and most (21/28, 75%) reported a goal of methamphetamine abstinence. Most (17/28, 61%) were receiving medication for opioid use disorder (14/28, 50% buprenorphine; 2/28, 7% injectable naltrexone; and 1/28, 4% methadone). In total, 4% (1/28) of the participants were prescribed an amphetamine medication. Per the EHR, most participants (22/28, 79%) had a co-occurring non-methamphetamine substance use disorder (most commonly opioid use disorder), and most (25/28, 89%) had a co-occurring mental health disorder (most commonly anxiety and depressive disorders; see Multimedia Appendix 1 for specific diagnoses). The Fisher exact tests indicated that there were no significant differences in these measures between participants who did and did not complete the welcome phase (all *P*>.05), with 1 exception: participants who completed the welcome phase more often reported 15 to 30 days of methamphetamine use in the past 30 days at baseline, whereas participants who did not complete the welcome phase were more likely to have fewer days of methamphetamine use (5-14 days; *P*=.02).

Table 2 provides additional descriptive information for the 86% (24/28) of participants who completed the baseline questionnaires. As shown in Table 2, most of these participants (21/24, 88%) reported DSM-5 criteria consistent with severe methamphetamine use disorder, and most reported consuming methamphetamine through smoking (19/24, 79%) or injection (12/24, 50%). Nearly all (23/24, 96%) reported using cannabis or at least one other illicit drug in addition to methamphetamine and other nonprescribed amphetamines, and most (19/24, 79%) reported using tobacco. Nearly all (22/24, 92%) had Medicaid insurance, and most (17/24, 71%) received Supplemental Nutrition Assistance Program benefits. Most (16/21, 76%) were not employed, and most reported housing insecurity (12/22, 55%) and financial insecurity (15/24, 62%). Many (10/24, 42%) reported a lack of transportation, and several (5/23, 22%) reported being incarcerated or involved with the criminal legal system within the past year. There were no significant differences in these measures between participants who did and did not complete the welcome phase (all *P*>.05), with 1 exception: participants who did not complete the welcome phase were more likely to report using cocaine (4/10, 40%) than participants who completed the welcome phase (0/14, 0%; *P*=.02).

**Table 1:** Characteristics of all consented participants (n=28).^a^

Characteristics			All participants who consented for the study (n=28), n (%)		Participants who consented and completed the welcome phase (n=15), n (%)		Participants who consented but did not complete the welcome phase (n=13), n (%)		*P* value
**Age range (years)**									.42
	18-29	5 (18)		4 (27)		1 (8)			
	30-45	9 (32)		5 (33)		4 (31)			
	46-64	14 (50)		6 (40)		8 (62)			
**Gender**									.33
	Woman	5 (18)		4 (27)		1 (8)			
	Man	23 (82)		11 (73)		12 (92)			
**Race**									.37
	American Indian or Alaska Native	1 (4)		0 (0)		1 (8)			
	Black or African American	3 (11)		2 (13)		1 (8)			
	Hispanic, Latino, Latina, or Spanish origin	4 (14)		1 (7)		3 (23)			
	Middle Eastern or North African	1 (4)		1 (7)		0 (0)			
	White or Caucasian	16 (57)		8 (53)		8 (62)			
	More than one race	3 (11)		3 (20)		0 (0)			
**Marital status**									.29
	Single, divorced, or widowed	22 (79)		10 (67)		12 (92)			
	Married or committed relationship	3 (11)		2 (13)		1 (8)			
	Other or unknown marital status	3 (11)		3 (20)		0 (0)			
Sexual orientation minority group			11 (39)		8 (53)		3 (23)		.14
**Past–30-day methamphetamine use**									.02
	5-9 days	4 (14)		1 (7)		3 (23)			
	10-14 days	4 (14)		1 (7)		3 (23)			
	15-20 days	7 (25)		7 (47)		0 (0)			
	21-30 days	13 (46)		6 (40)		7 (54)			
**Methamphetamine goal**									>.99
	Abstinence	21 (75)		11 (73)		10 (77)			
	Nonabstinent reduction	7 (25)		4 (27)		3 (23)			
**Prescribed medications for opioid use disorder**									.46
	Buprenorphine	14 (50)		5 (33)		9 (69)			
	Injectable naltrexone	2 (7)		2 (13)		0 (0)			
	Methadone	1 (4)		1 (7)		0 (0)			
	None	11 (39)		7 (47)		4 (31)			
**Other EHR^b^-based substance use and mental health disorder measures**									
	Prescribed amphetamine medication	1 (4)		1 (7)		0 (0)		>.99	
	Co-occurring nonamphetamine SUD^c^ (per EHR)	22 (79)		12 (80)		10 (77)		>.99	
	Co-occurring mental health disorder (per EHR)	25 (89)		15 (100)		10 (77)		.09	

^a^*P* values were obtained using Fisher exact tests and reflect differences between participants who completed the welcome phase and those who did not complete the welcome phase.

^b^EHR: electronic health record.

^c^SUD: substance use disorder.

**Table 2:** Characteristics of participants who completed the baseline assessment (n=24).^a^

Characteristics		All participants who completed the baseline assessment (n=24), n (%)	Participants who completed the baseline the assessment and completed the welcome phase (n=14), n (%)	Participants who completed the baseline assessment but did not complete the welcome phase (n=10), n (%)	*P* value	
**Methamphetamine use disorder symptom severity**						.70
	None (0-1 criteria)	1 (4)	0 (0)	1 (10)		
	Mild (2-3 criteria)	2 (8)	1 (7)	1 (10)		
	Moderate (4-5 criteria)	0 (0)	0 (0)	0 (0)		
	Severe (6-11 criteria)	21 (88)	13 (93)	8 (80)		
**Routes of methamphetamine administration**						
	Smoked	19 (79)	10 (71)	9 (90)	.36	
	Injected	12 (50)	7 (50)	5 (50)	>.99	
	Orally ingested	7 (29)	4 (29)	3 (30)	>.99	
	Snorted	9 (38)	6 (43)	3 (30)	.68	
	Other	2 (8)	2 (14)	0 (0)	.49	
**Other substances used in the past 30 days**						
	Alcohol	10 (42)	5 (36)	5 (50)	.68	
	Cannabis	13 (54)	7 (50)	6 (60)	.70	
	Cocaine	4 (17)	0 (0)	4 (40)	.02	
	Hallucinogens	2 (8)	0 (0)	2 (20)	.16	
	Heroin or other nonprescribed opioids	11 (46)	5 (36)	6 (60)	.41	
	Inhalants	1 (4)	0 (0)	1 (10)	.42	
	Ecstasy or molly	3 (12)	0 (0)	3 (30)	.06	
	PCP^b^	2 (8)	0 (0)	2 (20)	.16	
	Other synthetic drugs	2 (8)	1 (7)	1 (10)	>.99	
	Sedatives	9 (38)	4 (29)	5 (50)	.40	
	Other drugs	1 (4)	1 (7)	0 (0)	>.99	
	Any nonamphetamine drug listed above	23 (96)	13 (93)	10 (100)	>.99	
	Tobacco	19 (79)	11 (79)	8 (80)	>.99	
**Benefits**						
	Medicaid	22 (92)	12 (86)	10 (100)	.49	
	Disability	6 (25)	4 (29)	2 (20)	>.99	
	Social security	7 (29)	3 (21)	4 (40)	.39	
	Welfare	2 (8)	2 (14)	0 (0)	.49	
	Supplemental Nutrition Assistance Program	17 (71)	9 (64)	8 (80)	.65	
**Other baseline questionnaire measures**						
	Unhoused or in temporary or transitional housing	5 (21)	1 (7)	4 (40)	.12	
	Housing insecurity^c^	12 (55)	6 (46)	6 (67)	.41	
	Employed^c^	5 (24)	3 (25)	2 (22)	>.99	
	Lack of transportation	10 (42)	7 (50)	3 (30)	.42	
	Financial insecurity^d^	15 (62)	7 (50)	8 (80)	.21	
	Past-year jail or criminal justice system involvement^c^	5 (22)	3 (21)	2 (22)	>.99	

^a^*P* values were obtained using Fisher exact tests and reflect differences between participants who completed the welcome phase and those who did not complete the welcome phase.

^b^PCP: phencyclidine.

^c^In the subgroup of 14 participants who completed the welcome phase and the baseline survey, there was 1 (7%) participant who did not provide an answer about housing security and 2 (14%) who did not provide an answer to the question about employment. In the subgroup of 10 participants who did not complete the welcome phase but completed the baseline survey, there was 1 (10%) participant who did not provide an answer to the question about housing security, 1 (10%) who did not provide an answer to the question about employment, and 1 (10%) who did not provide an answer to the question about past-year jail or criminal justice system involvement.

^d^Financial insecurity was defined as the self-reported inability to pay for at least 2 of the following things when needed in the previous year: food, clothing, utilities, childcare, medicine or health care, phone, or other.

### Engagement

Of the 15 participants who completed the welcome phase and had access to the primary intervention, 1 (7%) requested early disenrollment citing low motivation to continue addressing their methamphetamine use and a lack of interest in continuing the intervention.

Other engagement metrics for the 54% (15/28) of participants who had access to the intervention are listed in Table 3. Rates of substance test completion (as a percentage of all tests requested by the app) varied across participants, ranging from 10% (3/29) to 94% (17/18) of the requested tests completed, with a mean of 35% (SD 27%; Table 3). On average, the rate of verified methamphetamine and amphetamine abstinence out of all substance tests requested by the smartphone app was 12% (SD 22%); across participants, this ranged from 0% to 94% (16/17) of the tests requested. On average, the rate of verified methamphetamine and amphetamine abstinence out of the substance tests that were completed by participants was 31% (SD 37%); across participants, this ranged from 0% to 100% (5/5) of the tests completed. Participants completed a mean of 5.6 (SD 6.5; range 0-24) calls with a CM guide out of the 12 encouraged. Participants completed a mean of 11.5 (SD 11.2; range 0-35) CBT modules out of the 35 available. Participants earned a mean of US $98.77 (SD US $60.38; range US $37-$236.38) in intervention rewards. After including compensation for research assessments, participants earned a mean of US $117.44 (SD US $56.93; range US $37-US $246.38) in total study-related compensation (Table 3).

**Table 3:** Engagement with the intervention among the participants who completed the welcome phase and received the intervention (n=15).

Engagement metric	Values, mean (SD)	Values, median (IQR; range)
Number of substance tests prompted by the smartphone app	24.9 (4.0)	25 (23-28; 17-30)
Number of substance tests completed	8.9 (7.1)	6 (3.5-11; 2-26)
Number of substance tests showing recent methamphetamine and amphetamine abstinence	2.7 (4.1)	2 (0-3; 0-16)
Number of substance tests that were invalid	0.5 (0.5)	1 (0-1; 0-1)
Substance tests that were completed out of those prompted (%)	35 (27)	24 (16-41; 10-94)
Substance tests showing recent methamphetamine and amphetamine abstinence out of those prompted by the smartphone app (%)	12 (22)	7 (0-13; 0-89)
Substance tests showing recent methamphetamine and amphetamine abstinence out of those completed (%)	31 (37)	10 (0-61; 0-100)
CM^a^ guide calls completed^b^	5.6 (6.5)	4 (0.5-8; 0-24)
CBT^c^ modules completed^d^	11.5 (11.2)	7 (4.5-17; 0-35)
Rewards earned (intervention only; US $)^e^	98.77 (60.38)	91 (48.50-128.92; 37-236.38)
Rewards earned (research assessments; US $)^e^	18.67 (16.63)	10 (10-27.50; 0-45)
Rewards earned (total; US $)^e^	117.44 (55.9)	107.04 (85.95-138.92; 37-246.38)

^a^CM: contingency management.

^b^Participants were encouraged to complete 1 CM guide call per week for the 12-week program but could complete additional coaching calls as desired.

^c^CBT: cognitive behavioral therapy.

^d^A total of 35 CBT modules were available.

^e^Participants could earn up to US $45 for completing research assessments plus US $280 in intervention rewards (US $325 total) if they enrolled before March 2022 or US $420 (US $465 total) if they enrolled in March 2022 or later.

### Usability

Table 4 summarizes the ratings for the intervention’s ease of use, satisfaction, and usefulness as rated by the 80% (12/15) of participants who received the intervention and completed the midintervention assessment. Participants generally expressed moderate to strong agreement with statements expressing the ease of use and satisfaction, reflecting favorable usability ratings. Participants generally expressed neutrality, moderate agreement, or strong agreement with statements reflecting the program’s usefulness for their health and well-being or the program’s ability to improve access to health care services. Participants expressed moderate to strong agreement with statements reflecting their comfort and confidence in using the program to communicate with their CM guide. No participants gave negative ratings (ie, “disagree” statements in Table 4) regarding the intervention’s ease of use, satisfaction, or usefulness.

Table 5 summarizes participants’ satisfaction with the amount of time it took to participate in the intervention on a day-to-day basis and their satisfaction with the duration of the intervention, as rated by the 73% (11/15) of participants who received the intervention and answered these questions on the midintervention questionnaire. A total of 91% (10/11) of the participants who completed the midintervention questionnaire reported that, on a day-to-day basis, the amount of time it took to participate in the program was “just right.” A total of 45% (5/11) of the participants reported that the overall 3-month duration of the program was “a little too short” or “much too short,” and 45% (5/11) of the participants reported that the 3-month duration was “just right.”

**Table 4:** Intervention usability ratings as reported on the modified mobile health (mHealth) App Usability Questionnaire at intervention week 6 (n=12).

Modified mHealth App Usability Questionnaire item			Strongly agree, n (%)		Agree, n (%)		Somewhat agree, n (%)		Neither agree nor disagree, n (%)		Somewhat disagree, n (%)		Disagree, n (%)		Strongly disagree, n (%)
**Ease-of-use questions**															
	The program was easy to use.	5 (42)		6 (50)		1 (8)		0 (0)		0 (0)		0 (0)		0 (0)	
	It was easy for me to learn to use the program.	4 (33)		7 (58)		1 (8)		0 (0)		0 (0)		0 (0)		0 (0)	
	I like the program.	6 (50)		6 (50)		0 (0)		0 (0)		0 (0)		0 (0)		0 (0)	
	The program was well organized, so I could easily find the information I needed.^a^	3 (27)		7 (64)		1 (9)		0 (0)		0 (0)		0 (0)		0 (0)	
	I feel capable of using this program.	7 (58)		4 (33)		1 (8)		0 (0)		0 (0)		0 (0)		0 (0)	
**Satisfaction questions**															
	I would use this program again.	7 (58)		4 (33)		1 (8)		0 (0)		0 (0)		0 (0)		0 (0)	
	Overall, I am satisfied with the program.	6 (50)		5 (42)		1 (8)		0 (0)		0 (0)		0 (0)		0 (0)	
	The program is an acceptable way to receive help with substance use.	5 (42)		5 (42)		2 (17)		0 (0)		0 (0)		0 (0)		0 (0)	
	The program does what I expected it to.	3 (25)		8 (67)		0 (0)		1 (8)		0 (0)		0 (0)		0 (0)	
**Usefulness questions**															
	The program would be useful for my health and well-being.	5 (42)		7 (58)		0 (0)		0 (0)		0 (0)		0 (0)		0 (0)	
	The program improved my access to health care services.	3 (25)		2 (17)		3 (25)		4 (33)		0 (0)		0 (0)		0 (0)	
	The program helped me manage my substance use effectively.	2 (17)		4 (33)		3 (25)		3 (25)		0 (0)		0 (0)		0 (0)	
	I felt confident that any information I sent to my recovery coach using the app would be received.	7 (58)		3 (25)		1 (8)		1 (8)		0 (0)		0 (0)		0 (0)	
	I felt comfortable communicating with my recovery coach using the app.	5 (42)		4 (33)		2 (17)		1 (8)		0 (0)		0 (0)		0 (0)	

^a^One participant did not answer the question.

**Table 5:** Ratings of the intervention’s time requirements and duration, obtained by questionnaires at intervention week 6 (n=11).

	Much too short, n (%)	A little too short, n (%)	Just right, n (%)	A little too long, n (%)	Much too long, n (%)
On a day-by-day basis, the amount of time it takes to participate in the program is:	0 (0)	1 (9)	10 (91)	0 (0)	0 (0)
The 3-month duration of this program seems:	1 (9)	4 (36)	5 (45)	1 (9)	0 (0)

### Supplemental Clinical Outcomes

Multimedia Appendix 1 provides results describing changes in supplemental clinical outcome measures from baseline to 12 weeks for the 39% (11/28) of participants who received the intervention and completed measures at baseline and the 12-week follow-up. Results suggest that participants reported significantly fewer methamphetamine use disorder criteria at the 12-week follow-up (mean 7.00, SD 3.61) compared with baseline (mean 8.73, SD 2.76; *P*=.04; Cohen *d*=−0.54, 95% CI −1.04 to −0.03). There were no significant changes in depression screenings (*P*=.78), methamphetamine abstinence self-efficacy (*P*=.27), or social support (*P*=.17).

## Discussion

### Principal Findings

This pilot study evaluated the feasibility of offering a fully remote, modified version of a commercially available mHealth CM program to patients who used methamphetamine and who were receiving treatment within a health system that did not provide other CM programs. We found that just over half (15/28, 54%) of the participants who enrolled in the study were able to complete the “welcome phase” activities required for starting the primary intervention. Among those who completed the welcome phase, we found variable rates of engagement with substance testing, CM guide calls, and self-paced CBT modules. Rates of methamphetamine and amphetamine abstinence verified via substance tests were generally low, although participants noted fewer methamphetamine use disorder criteria at the end of the study. Participants gave favorable ratings for ease of use and satisfaction with the intervention and had a mixture of favorable and neutral ratings for the intervention’s usefulness. No participants gave unfavorable ratings for the ease of use, satisfaction, or usefulness. Together, these results suggest that fully remote CM programs may be feasible, engaging, and usable for some patients who use methamphetamine. Nonetheless, some patients may struggle to engage with fully remote CM, suggesting that additional modifications or alternatives may be beneficial, as discussed more in the following sections.

### Clinical Implications

#### Uptake With the Fully Remote Intervention Was a Barrier for Some Patients Who Use Methamphetamine

Just under half (13/28, 46%) of the participants were not able to complete the necessary tasks for onboarding to the intervention as it was protocolized for this study, potentially because of fatigue associated with both the research activities (ie, screening, consent, and baseline assessment) and the intervention onboarding activities (ie, contacting the mHealth program and completing the welcome phase activities). This limited the extent to which participants who enrolled in the study were able to receive the primary intervention. For future implementations of remotely delivered CM, barriers that limit intervention uptake will be critical to address as they can limit the reach and impact of interventions. It is possible that the procedures developed for this study (eg, completing screening, consent, and baseline assessments by phone and transferring patients to the mHealth vendor to initiate the welcome phase, as well as the monetary reward values selected for the pilot program) and the monetary values that were used for the onboarding period could have limited completion of the onboarding procedures. Thus, more streamlined enrollment processes and higher reward values for the welcome phase activities could have increased uptake.

Regular methamphetamine use is known to undermine many cognitive domains [29] and may have hindered participants’ ability to engage with treatment. Fluctuations in motivation over time may also have been a contributing barrier [30]. Uptake could have also been limited by high rates of psychosocial challenges in addition to frequent methamphetamine use (eg, high rates of non-methamphetamine substance use disorders, mental health disorders, and housing and economic insecurities). These factors likely imposed barriers that limited participants’ capacity to fully engage and can be explored more thoroughly in future qualitative studies.

In subgroup analyses, participants who did not complete the welcome phase used methamphetamine less frequently at baseline and were more likely to report past–30-day cocaine use at baseline. Thus, it is also possible that this study’s choice not to reward cocaine abstinence decreased the usefulness of the intervention for some participants if cocaine was also a significant concern. Future studies addressing methamphetamine may choose to also incentivize cocaine abstinence to make the intervention more relevant to individuals who also use cocaine.

For remotely delivered mHealth interventions with people who use methamphetamine, additional human-to-human connection may be needed to help facilitate intervention uptake, particularly during the early stages of the intervention. Of note, this study was designed to be fully remote with no in-person contact, partly because of concerns about COVID-19 infection risk and pandemic-related workflow challenges that disrupted usual workflows across the participating clinics. However, hybrid models blending mHealth interventions with in-person support might be viable and preferable to support participants in initiating engagement, particularly for those who use methamphetamine. For example, a previous study evaluating the DynamiCare program in patients with stimulant and opioid use disorders receiving buprenorphine treatment [12] rewarded in-clinic activities (eg, clinic visits and drug abstinence verified via urine samples collected at the clinic) and, thus, used the mHealth program to reinforce in-person clinical activities rather than as a fully remote intervention. In addition, motivational interviewing or more explicit shared decision-making steps during the referral process may increase uptake. Alternatively, patient navigation models could potentially be combined with CM to assist participants with initial uptake [31], although we are not aware of studies that have combined patient navigation with mHealth interventions designed for people who use methamphetamine.

Other studies have used remote substance testing through DynamiCare when targeting alcohol [10] and nicotine abstinence [15-17], and it is possible that uptake with remote substance testing and videoconferencing may be more feasible in populations of individuals who are addressing their alcohol and nicotine use than in populations addressing their methamphetamine use. Studies have shown that people who use methamphetamine have a significantly decreased likelihood of initiating and continuing other treatments (eg, programs that offer medications for opioid use disorder), potentially because of challenges associated with methamphetamine use as well as the limited availability of evidence-based treatments for methamphetamine use disorder [32-34]. A study testing in-person CM for methamphetamine found that only 65% of eligible participants completed the initial substance tests and in-person clinic visits required to receive the primary intervention (analogous to this study’s “uptake” measure) [35]. These studies illustrate the challenges with treatment initiation and engagement in patients seeking methamphetamine treatment. Thus, the 54% (15/28) uptake observed in this study may largely reflect challenges that are common across treatments with populations who use methamphetamine rather than reflecting limitations that are inherent to the modality of the intervention. This study sample consisted of individuals with high rates of psychosocial challenges, which may make it difficult to participate in any treatment modality. Importantly, participants who were able to successfully initiate and engage with the intervention consistently provided high ease-of-use and satisfaction ratings. These results provide some reassurance that the mechanics of using the smartphone-based intervention were unlikely to have been a significant barrier to participants who received the intervention; however, less is known about whether this was a barrier for those who did not receive the intervention.

#### The Intervention Yielded Variable Rates of Substance Testing and Low Rates of Verified Methamphetamine Abstinence

Among participants who received the primary intervention, there were variable rates of substance test completion when requested by the smartphone app and typically low rates of verified methamphetamine and amphetamine abstinence. There are several modifiable factors that could have led to these outcomes. For example, some participants may have required a longer intervention period (eg, 12 months instead of 12 weeks) to integrate behavioral and environmental changes that, in combination with CM, could support sustained methamphetamine abstinence. The potential benefits of a longer intervention period are also reflected in the midintervention usability ratings, where 45% (5/11) of participants who completed the questionnaire reported that the 12-week duration of the intervention seemed shorter than they would have preferred. The rates of verified methamphetamine and amphetamine abstinence may have been higher if greater financial incentives had been offered for verified abstinence, especially considering the increased availability of inexpensive methamphetamine throughout the region during the time the intervention was offered.

It is also plausible that participants sometimes skipped substance tests when they had recently used methamphetamine given that there would be no reward for completing such tests. Thus, the low rates of testing could have been partially reflective of the low rates of recent methamphetamine abstinence. Offering smaller rewards for completing substance tests—regardless of the test result—could potentially increase engagement with substance testing for participants who struggle with continued methamphetamine use. Although such rewards would not be expected to reinforce abstinence, they could reinforce engagement with the intervention as an initial step toward a longer-term goal of reducing or abstaining from methamphetamine use. Alternatively, interventions could specifically target buprenorphine adherence for patients who use methamphetamine and have co-occurring opioid use disorder as there is evidence indicating that people who use methamphetamine have lower retention in buprenorphine treatment for opioid use disorder and that those who remain in buprenorphine treatment demonstrate reduced methamphetamine use [32,33]. For example, interventions could administer rewards for videos in which patients demonstrate themselves taking buprenorphine every day or completing biological substance tests that indicate the presence of buprenorphine in the body, with additional rewards available for methamphetamine abstinence.

Several participants anecdotally mentioned that they did not complete substance tests because they forgot to take tests with them when leaving their home despite instructions during the welcome phase to carry substance tests with them whenever they left home and similar encouragement from CM guides. This barrier may be particularly salient for randomly scheduled prompts to complete substance tests (used in this intervention), in contrast to other CM interventions where substance testing times are scheduled in advance or where CM rewards are disbursed for attending scheduled clinic visits. Future interventions that use randomly scheduled substance testing could offer focused skills training that aims to help participants identify ways to remember to bring substance tests with them when leaving home. Similarly, such interventions could also offer “travel kits” with substance testing supplies that participants can keep in a backpack, purse, or car so that they are more likely to have substance testing supplies with them when they are away from home.

Nonetheless, even with significant changes, many participants will likely struggle to abstain from methamphetamine, particularly when symptoms are consistent with severe methamphetamine use disorder. Despite self-reporting a goal of reducing or abstaining from methamphetamine use initially, the motivation to pursue such goals and biopsychosocial vulnerabilities that inhibit one’s ability to achieve these goals can fluctuate substantially over time [36]. Therefore, future iterations of mHealth CM interventions could be designed to support participant engagement and benefit with the anticipation that participants’ motivation and ability to achieve and maintain significant changes in methamphetamine use will likely vary considerably. Future interventions could incorporate motivational interviewing strategies to enhance motivation and commitment early in the intervention. Future interventions could also offer greater rewards aimed at promoting engagement for participants who do not achieve methamphetamine abstinence yet take active steps toward change or improved health, for example, by increasing rewards for activities that do not require abstinence (CM guide calls and CBT modules) or offering financial rewards for attending outpatient medical, mental health, and addiction treatment appointments [12]. Future interventions could also incorporate rewards for activities that could potentially enhance self-reflection, goal-setting, and self-tracking activities aimed at increasing motivation and commitment, such as mindfulness, self-monitoring, and goal-setting activities [14].

### Research Implications

This pilot study also provides insights that can help inform the designs of future, larger, and more rigorous clinical trials. For example, future studies of remotely delivered CM may wish to include a “run-in” or “prebaseline” period if they wish to maximize power for testing the efficacy of the intervention. During such a run-in period, participants could be required to demonstrate an initial ability to adhere to the study procedures (eg, complete videoconference calls and practice tests) before being randomized to a study intervention condition. Similar procedures have been used in previous studies of mHealth- and in-person–based CM [16,35] and resulted in the exclusion of participants who were unable to achieve a minimum level of engagement with the intervention, which in turn could increase statistical power to detect positive outcomes associated with the intervention. However, including a run-in period could also impose limitations for understanding the impact of an intervention within real-world health systems given that many patients who struggle with intervention uptake would be excluded from studies that use a run-in design. This could yield a sample of patients who may be more capable of engaging with interventions relative to the population of patients who would be referred to the intervention within a health system.

To potentially improve uptake and engagement, future studies could also incorporate greater involvement from the target population in setting up or modifying the treatment program. Additional involvement from referring and treating clinics and clinicians could also be incorporated into the intervention, for example, by offering substance testing on-site at clinics for patients who struggle with remote testing. On-site “enrollment specialists” have been employed in mHealth interventions to help facilitate more human connection with participants and help troubleshoot barriers to uptake and engagement [37]. Future studies should specifically target increasing uptake and engagement with mHealth and CM interventions as meaningful outcomes given the robust evidence for the efficacy of CM for people with stimulant use disorders [4].

Finally, future studies may implement processes to streamline the steps involved for onboarding, including the process of referring, consenting, and completing the welcome phase activities. On average, it took more than a month for participants in this study to complete these tasks, and it is possible that a long waiting period could negatively affect motivation for engaging with the intervention for some individuals [38].

### Limitations and Strengths

This study has noteworthy limitations. It was a single-arm pilot study that did not include randomization or a control condition, and thus, by design, it was unable to test the effectiveness or efficacy of the intervention. The small sample size limits the precision of the findings and our ability to perform subgroup analyses. During the screening calls, methamphetamine use was assessed using self-report rather than biological verification, although patient-reported methamphetamine use has been shown to correspond with biological testing results [39]. Although some patients completed substance testing as part of their treatment within the health system, these tests were not reliably available and, thus, were not used for this research. The study was conducted within a single multisite academic health system with participants who had high rates of co-occurring substance use and mental health disorders; thus, this sample reflects a population with considerable treatment needs, and the findings may not be generalizable to other settings and populations. Smartphone literacy was not assessed and could have limited participation for some individuals. Aspects of the intervention protocol were modified to fit the needs and desires of clinicians in the clinical settings where the pilot was conducted; thus, the results may not be directly comparable with those of other studies or settings that use DynamiCare. For example, this intervention differed from other DynamiCare studies in that the procedures were fully remote, in-clinic visits were not rewarded, and CM rewards were not conditioned on abstinence from cocaine and other drugs. In addition, as it was a pilot study, the intervention was only 12 weeks long, and reward values for some activities were more limited compared with more typical DynamiCare protocols. This intervention was modified midway through the study with the goal of increasing engagement, and the results should be interpreted accordingly. Some analyses excluded participants who had missing data on the baseline questionnaire (4/28, 14% of participants who enrolled in the study) or missing data on the usability questionnaire (3/15, 20% of participants who received the intervention).

This study also has noteworthy strengths. The fully remote design allowed participants to take part in a CM intervention even under circumstances that would usually limit engagement (eg, if they lacked transportation, left the metropolitan area, or were sick with COVID-19). The fully remote design also represented a realistic scenario for how CM interventions could be implemented in a scalable way, especially in clinics that lack the staffing, resources, and workflows to maintain CM programs (eg, most primary care settings). Thus, the fully remote design offered an important starting point for identifying how the intervention worked when offered in relative isolation from other services. This helped identify where additional in-person support may be needed. This was the first study to prospectively evaluate the DynamiCare intervention for addressing methamphetamine use. This is notable given that evidence-based behavioral interventions for methamphetamine are often unavailable in most real-world settings, and there are currently no FDA-approved medications for methamphetamine use disorder. In addition, an important feature of the study design is that the procedures created minimal disruption to clinical workflows, and little effort was required from clinicians to make the CM program available to their patients.

### Conclusions

Leveraging digital technology to deliver CM may be a viable long-term strategy facilitating widespread implementation of CM. This study found that a fully remote CM program could be feasibly offered within a large health care system where in-person CM programming has been difficult to implement. The use of an mHealth platform and fully remote procedures allowed the CM intervention to become readily available in multiple clinics in a manner that may have been considerably less time-consuming to implement compared with traditional face-to-face service delivery models. Many patient participants struggled to complete the required welcome phase activities (completing 2 videoconference calls and 2 practice saliva-based substance tests) after having completed research onboarding activities (screening, informed consent, and baseline survey), limiting intervention uptake. Participants who achieved uptake reported high usability and had variable rates of engagement with the intervention components. Future studies may focus on approaches to increasing intervention uptake and engagement for people who use methamphetamine—a population that often has considerable unmet needs in the areas of addiction, mental health, medical care, and socioeconomic hardship. Potential modifications could include offers of enhanced support to increase early engagement, substantial shortening of the enrollment period, further optimization of the reward schedule, mechanisms for offering more incentives earlier in the engagement period, and the implementation of rewards for additional non–abstinence-based outcomes that could facilitate treatment engagement. These and other adjustments can be incorporated into future studies on mHealth-delivered CM for people who use methamphetamine.

## Appendix

Multimedia Appendix 1 Supplemental descriptive analyses, including electronic health record–documented substance use and mental health disorders at baseline, supplementary clinical outcome measures, and the contingency management reward schedule used in the intervention.

## Abbreviations

CBT: cognitive behavioral therapy
CM: contingency management
DSM-5: Diagnostic and Statistical Manual of Mental Disorders, Fifth Edition
EHR: electronic health record
FDA: Food and Drug Administration
mHealth: mobile health
REDCap: Research Electronic Data Capture
