# Supplementary material for: Feasibility, Engagement, and Usability of a Remote, Smartphone-Based Contingency Management Program as a Treatment Add-On for Patients Who Use Methamphetamine: Single-Arm Pilot Study
Source: JMIR Form Res. 2023 Jul 6;7:e47516. doi: 10.2196/47516 (PMC10360016; doi:10.2196/47516)
Supplement: Multimedia Appendix 1 [file formative_v7i1e47516_app1.docx]

| Table S1 *Baseline substance use and mental health disorders, per electronic health records (EHR) over the year prior to study enrollment.* | | | | | | | | |
| --- | --- | --- | --- | --- | --- | --- | --- | --- |
|  |  | All patients who consented for the study  (n = 28) | | Patients who consented and received the intervention  (n = 15) | | Patients who consented but did not receive the intervention  (n = 13) | |  |
|  |  | N | (%) | N | (%) | N | (%) | p-value |
| **Substance Use Disorders** | Alcohol | 6 | (21%) | 5 | (33%) | 1 | (8%) | 0.17 |
|  | Cannabis | 5 | (18%) | 3 | (20%) | 2 | (15%) | 1.00 |
|  | Cocaine | 4 | (14%) | 2 | (13%) | 2 | (15%) | 1.00 |
|  | Opioid | 18 | (64%) | 9 | (60%) | 9 | (69%) | 0.71 |
|  | Sedatives | 5 | (18%) | 4 | (27%) | 1 | (8%) | 0.33 |
|  | Other drug use disorder | 12 | (43%) | 8 | (53%) | 4 | (31%) | 0.28 |
| **Mental Health Disorders** | Adjustment disorder | 2 | (7%) | 1 | (7%) | 1 | (8%) | 1.00 |
|  | Anxiety disorder | 19 | (68%) | 11 | (73%) | 8 | (62%) | 0.69 |
|  | Attention deficit / hyperactivity disorder | 4 | (14%) | 2 | (13%) | 2 | (15%) | 1.00 |
|  | Bipolar disorder | 7 | (25%) | 3 | (20%) | 4 | (31%) | 0.67 |
|  | Delusional disorder | 3 | (11%) | 1 | (7%) | 2 | (15%) | 0.58 |
|  | Depressive disorder | 17 | (61%) | 10 | (67%) | 7 | (54%) | 0.70 |
|  | Eating disorder | 2 | (7%) | 1 | (7%) | 1 | (8%) | 1.00 |
|  | Post-traumatic stress disorder | 7 | (25%) | 2 | (13%) | 5 | (38%) | 0.20 |
|  | Sleep disorder | 2 | (7%) | 1 | (7%) | 1 | (8%) | 1.00 |
|  | Schizophrenia spectrum disorder | 1 | (4%) | 1 | (7%) | 0 | (0%) | 1.00 |
|  | Other unspecified mental disorder | 4 | (14%) | 1 | (7%) | 3 | (23%) | 0.31 |
| *Note*. P-values were obtained using Fisher's exact tests and reflect differences between patients who received the intervention and patients who did not receive the intervention. | | | | | | | | |

# Supplemental Clinical Measures

Clinical measures were administered at baseline and 12-week follow-up (end of intervention). The table below provides mean and standard deviation values for these measures among the 11 participants who received the mHealth intervention and completed the baseline and 12-week follow-up assessments.

Measures included:

1. Methamphetamine use disorder symptoms, measured using the Substance Use Symptom Checklist [18]. The measure contains 11 items where participants self-reported the presence or absence of each of the 11 criteria for methamphetamine use disorder (MUD) based on the criteria defined by the Diagnostic and Statistical Manual of Mental Disorders 5^th^ edition (DSM-5) [19]. The number of MUD criteria patients endorsed was summed, yielding scores ranging from 0 to 11.
2. Depression screens, measured using the Patient Health Questionnaire-2 (PHQ-2) [20]. The measure contains 2 items where patients report the frequency in which they have experienced anhedonia and depressed mood within the past two weeks. Item responses range from “not at all” (0) to “nearly every day” (3) and are summed to yield scores ranging from 0 to 6. The measure has been shown to have good sensitivity and specificity for detecting depression [20]. The measure has also been shown to be sensitivity to detecting changes in depression symptoms over time [21].
3. Methamphetamine abstinence self-efficacy was measured using a single-item self-efficacy question [22]. Patients reported how confident they were that they would not use methamphetamine over the next 30 days, with confidence ratings ranging from “not at all” (1) to “extremely” (5).
4. Social support was measured using 4 psychometrically questions from the Medical Outcome Study Social Support Survey [23]. The questions ask how often the participant has someone who is available to provide different forms of social support (e.g., help doing chores if sick, suggestions for dealing with a personal problem, etc.). Response options range from “none of the time” (1) to “all of the time” (5) and are summed to provide total scores ranging from 4 to 20.

Differences between scores at baseline and 12-week follow-up were tested using paired sample t-tests. Cohen’s d statistics (and 95% CI’s) were estimated to reflect the standardized differences in mean scores from baseline to 12-week follow-up, expressed in standard deviation units.

Results are presented here as supplementary because they can help inform future trial designs (e.g., inform power analyses). However, they are not reported as primary results because of low power from the small sample and the primary study’s focus on feasibility, engagement, and usability rather than clinical effectiveness.

| Table S2 *Changes from baseline to 12-weeks among n = 11 patients who received the intervention and completed the baseline and 12-week assessments.* | | | | | | | | | |
| --- | --- | --- | --- | --- | --- | --- | --- | --- | --- |
|  | **Baseline** | | **12-week** | | **Difference** | | | | |
| **Measure (scale range)** | **M** | **(SD)** | **M** | **(SD)** | **d** | **(95% CI)** | | **p-value** | |
| DSM-5 MUD symptoms (0-11) | 8.73 | (2.76) | 7.00 | (3.61) | -0.54 | (-1.04, | 0.03) | | 0.04 |
| PHQ-2 depression screen (0-6) | 2.18 | (1.60) | 2.09 | (2.02) | 0.05 | (-0.43, | 0.33) | | 0.78 |
| Methamphetamine abstinence self-efficacy (1-5) | 3.18 | (1.17) | 2.82 | (1.17) | -0.31 | (-0.90, | 0.28) | | 0.27 |
| Social support (4-20) | 10.91 | (3.42) | 12.73 | (4.76) | 0.44 | (-0.23, | 1.10) | | 0.17 |
| *Note*. DSM-5=Diagnostic Manual of Mental Disorders 5th edition. MUD=methamphetamine use disorder. P-values were obtained using paired sample *t*-tests. | | | | | | | | | |

# Reward Schedule for Substance Testing

As patients provided consecutive tests indicating recent methamphetamine abstinence, the frequency of testing decreased. As the frequency of testing decreased, the reward values increased in a commensurate amount such that $16.85 in rewards was expected to be available per week for negative substance testing. (Note, however, that substance tests were prompted randomly and therefore the number of tests prompted per week could vary.)

The table below indicates how the frequency of testing and reward values changed with consecutive tests indicating recent methamphetamine abstinence.

When a patient provided a test that indicated recent methamphetamine abstinence, their streak moved up by 1.

When a substance test was missed/skipped or indicated recent methamphetamine use, their streak moved down by 2.

| **Streak** | **Saliva tests per week** | **Reward per saliva test** |
| --- | --- | --- |
| 0 | 2 | $8.42 |
| 1 | 1.95 | $8.64 |
| 2 | 1.9 | $8.87 |
| 3 | 1.85 | $9.11 |
| 4 | 1.8 | $9.36 |
| 5 | 1.75 | $9.63 |
| 6 | 1.7 | $9.91 |
| 7 | 1.65 | $10.21 |
| 8 | 1.6 | $10.53 |
| 9 | 1.55 | $10.87 |
| 10 | 1.5 | $11.23 |
| 11 | 1.45 | $11.62 |
| 12 | 1.4 | $12.03 |
| 13 | 1.35 | $12.48 |
| 14 | 1.3 | $12.96 |
| 15 | 1.25 | $13.48 |
| 16 | 1.2 | $14.04 |
| 17 | 1.15 | $14.65 |
| 18 | 1.1 | $15.31 |
| 19 | 1.05 | $16.04 |
| 20 | 1 | $16.85 |
| 21 | 1 | $16.85 |
